# Rigid Polyurethane Foams Modified with Biochar

**DOI:** 10.3390/ma14195616

**Published:** 2021-09-27

**Authors:** Katarzyna Uram, Maria Kurańska, Jacek Andrzejewski, Aleksander Prociak

**Affiliations:** 1 Department of Chemistry and Technology of Polymers, Faculty of Chemical Engineering and Technology, Cracow University of Technology, Warszawska 24, 31-155 Cracow, Poland; katarzyna.uram@doktorant.pk.edu.pl (K.U.); aleksander.prociak@pk.edu.pl (A.P.); 2 Polymer Processing Division, Faculty of Mechanical Engineering, Institute of Materials Technology, Poznan University of Technology, Piotrowo 3, 61-138 Poznan, Poland; jacek.andrzejewski@put.poznan.pl

**Keywords:** biochar, rigid polyurethane foams, thermal conductivity, polymer fillers, sustainability

## Abstract

This paper presents results of research on the preparation of biochar-modified rigid polyurethane foams that could be successfully used as thermal insulation materials. The biochar was introduced into polyurethane systems in an amount of up to 20 wt.%. As a result, foam cells became elongated in the direction of foam growth and their cross-sectional areas decreased. The filler-containing systems exhibited a reduction in their apparent densities of up to 20% compared to the unfilled system while maintaining a thermal conductivity of 25 mW/m·K. Biochar in rigid polyurethane foams improved their dimensional and thermal stability.

## 1. Introduction

In recent years, published studies have often concerned the sustainable development of polymeric materials. One approach of such works is to replace the mineral or petrochemical fillers with renewable ones. The advantage of bio-fillers is their low density, and the composites obtained with such additives can have better properties than those with mineral fillers [1]. The use of biomass as a filler is one way of solving environmental problems. Recently, plant-based fillers have been gaining popularity, and can be successfully used in the production of polyurethane composites [2]. Interestingly, the combustion of biofuels does not contribute to the greenhouse effect. This is due to the neutral conversion of carbon dioxide, which is conditioned by the renewal of lignocellulose biomass. In addition, biomass plays an important role in the circular economy [3,4].

Biochar (biocarbon, BioC) is a carbon material obtained by pyrolysis of biomass, which comes from inexpensive and sustainable sources. This material can be used as a carbon modifier and absorbing agent to remove metals from contaminated soil [5]. In addition, biochar is used as a raw material for the production of activated carbon [4]. Pyrolysis is a process of thermal decomposition that can take place in the temperature range from 300 to 800 °C and with total or partial lack of oxygen. As a result of this process, solid biochar, bio-oil and fuel gas are obtained. The biochar is characterized by a porous structure with a large specific area. Thanks to this structure, for example, it increases the adsorption capacity of biochar to retain moisture and nutrients in the soil. This method is an alternative in the use of agricultural and industrial waste [6]. There are studies in the literature on the use of biochar as a filler in different types of polymers. The use of biochar in poly(lactid acid) resulted in reduced abrasion and flammability of composites [7] or increased thermal stability during injection molding [8]. On the other hand, the use of biochar as a replacement for carbon fibers increased the tensile strength and impact strength of polycarbonate (PC) composites [9]. Another paper describes the use of biochar as part of polymer blends polycarbonate/acrylonitrile-butadiene-styrene (PC/ABS) [10]. Biochar added to PC causes a decrease in the glass transition temperature and degradation process of this polymer. However, when combined with PC/ABS, both the problem of excessive hydrolytic degradation and lowering of glass transition temperature in PC/biochar systems were effectively eliminated. These studies indicate that biocarbon can successfully replace other mineral fillers. 

Up to now, the use of the biochar-based fillers has been mostly focused on the thermoplastic polymers applications, while there are only a few examples of the BioC use in a reactive system, like epoxy [11,12,13,14] or unsaturated polyesters [15,16]. In most cases, BioC was used as a sustainable type of filler, usually leading to a slight improvement in the stiffness of composites. So far, examples of applications of BioC in polyurethane-based systems have been described very rarely. In the research conducted by Meng et al., BioC-modified porous polyurethanes were used as the filter components in a bioretention system [17]. 

Rigid polyurethane foams (RPUF) are obtained during the reaction of compounds with hydroxyl groups and compounds containing isocyanate groups. They have a porous chemical structure and can be successfully used as insulation materials. Such insulators are characterized by a low thermal conductivity coefficient and good mechanical properties and water resistance [18]. The cell structure and thermal insulation properties depend on the foaming process of the foam material. Not only the process conditions are responsible for the foaming process, but also a properly selected catalytic system or the reactivity of the ingredients used [19]. Adding filler to a polyurethane system can cause filler agglomerates to form and cause structural damage to the rigid polyurethane foams. In addition, the introduction of a filler increases the viscosity of the polyol premix, disturbing the foaming process [20]. 

Adding a filler to the polyurethane system can change the process. The growing concern for the environment raises the interest in polyurethane foams based on renewable sources. Every year, the polyurethane market grows, and thus generates more waste. Obtaining the foam in an ecological version, i.e., from renewable sources, may pose a lower threat to the environment [21,22]. A major challenge is to obtain rigid foams with good mechanical strength and, at the same time, low thermal conductivity. The addition of a filler component to the polyurethane system could improve the properties of foams. It is most advantageous to use fillers derived from natural sources because they are inexpensive. There are studies in which both powders and fibers are used as fillers. These include agricultural wastes (bagasse) [23], lignin [24,25,26,27], cellulose [28,29,30,31], carrot nanofibers [32], eucalyptus fibers [18]. Rafael de Avila Delucis et al. [33] used six different forest fillers in different amounts (1, 5 and 10 wt.%) in rigid polyurethane foams. They observed that at filler contents of more than 5%, a large number of filler agglomerates are formed outside the cell wall of the polymer, which deteriorates the compressive properties of foams. However, the use of forest fillers resulted in a homogeneous cell structure of polyurethane foams. Lady Jaharah Y. Jabber et al. [34] obtained rigid polyurethane foams with cellulose fibers (1, 3 and 5 wt.%) extracted from pineapple. They observed that the introduction of the filler decreased the mechanical strength of the foams. The decrease in the compressive strength was caused by the decrease of the apparent density of the polyurethane foams. Moreover, the addition of cellulose fibers can reduce the stiffness and thus cause the cells of the foam to break.

This article presents research on the modification of rigid polyurethane foams with biochar (BioC). Systems containing 3, 6, 9 and 20 wt.% BioC were prepared and examined in terms of cell structure, apparent density and thermal conductivity. Additionally, the mechanical and thermal properties of the systems were analyzed. 

## 2. Materials and Methods

### 2.1. Materials

Polyether polyol—Rokopol RF-551—having a hydroxyl value of ca. 420 mgKOH/g and a water content of 0.10 wt.% was supplied by PCC Rokita S.A. Polycat 218 produced by Evonik Industries AG was used as a catalyst. This is a reactive amine catalyst providing strong urea-reaction (blowing) catalysis. Niax silicone L-6915 supplied by Momentive Performance Materials Inc. was used as a stabilizer of the foam structure. The carbon dioxide generated during the reaction between water and isocyanate played a role of blowing agent. Polymeric methylene diphenyldiisocyanate was supplied by Minova Ekochem S.A.

**Figure 1:**
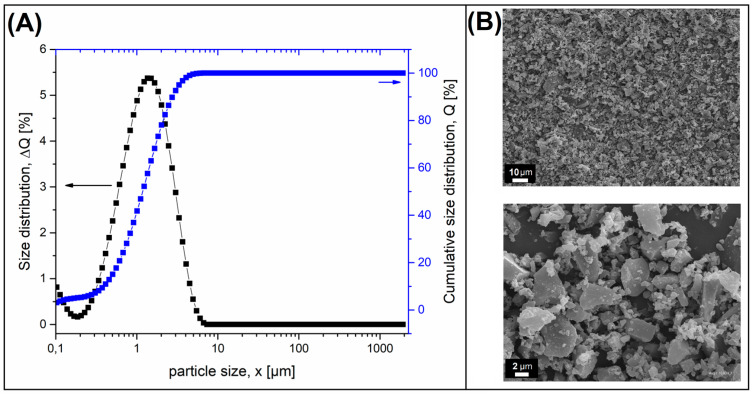
(**A**) Size distribution histograms and (**B**) SEM microphotograph of biochar (BioC) particles.

The biochar (BioC) filler used during this study was purchased from the company Fluid SA. For this particular type of BioC, the pyrolysis process was conducted at 650 °C, while the material itself was prepared on the basis of wood chips. The mean particle size in the untreated BioC was around 200 µm. Before the introduction to the polyol, the BioC filler was ball-milled for 24 h. The milling procedure reduces the average particle size. The size analysis, performed with the use of a laser particle sizer (Fritsch Analysette 22), indicates that the mean particle size is 1.5 µm, while 80% of the particles are within the size range from 0.18 to 3.5 µm. The chart presented in the Figure 1A collates the particle size distribution plots and cumulative size distribution plots. The appearance of the BioC particles after ball milling is presented in Figure 1B.

**Figure 2:**
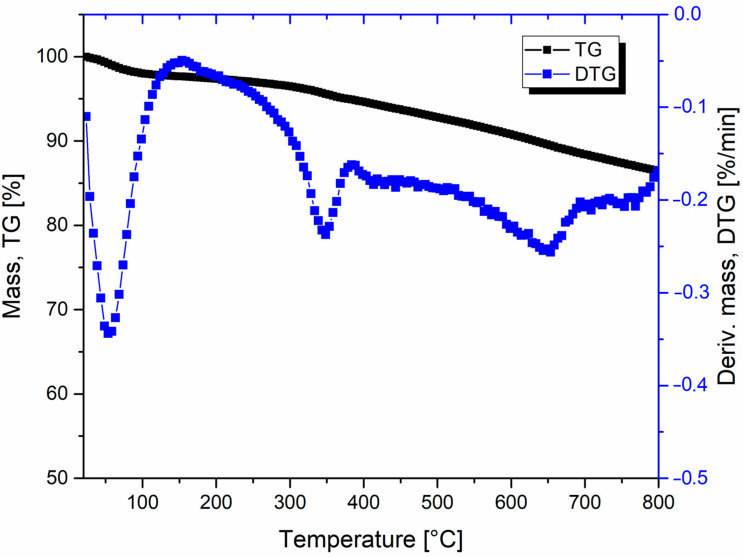
The results of the thermogravimetric analysis for pure BioC filler. Mass loss (TG) and derivative mass (DTG) plots are combined on one chart.

In order to verify the basic thermal properties of the BioC filler, the ball milled filler was subjected to preliminary TGA analysis, the results in the form of TG and DTG thermograms are presented in the Figure 2. Due to the fact that biochar is a biomass subjected to a thermal treatment process at about 650 °C, therefore thermal stability of BioC is a significant advantage of this type of material, compared to classic type of natural fillers like sawdust. It is important to note that the residual mass at the temperature of 800 °C reaches 87% of the original sample charge, which proves the high elemental carbon content. The loss of sample mass is divided into several stages, among which it is difficult to select one dominant. According to the research conducted so far, the decrease in the mass of biochar is related to the loss of moisture and the decomposition of organic groups [35,36].

### 2.2. Preparation of Rigid Polyurethane Foams

A reference rigid polyurethane foam and products modified with BioC were prepared by a single-step method. The polyol premix consisting of a polyol, catalyst, surfactant, blowing agent and biochar was mixed for 60 s. Next, the polyol premix and isocyanate were mixed for 6 s and poured into a mold. The amount of biochar was 3, 6, 9, and 20% of to the total mass of the polyol and isocyanate. Free rise foaming took place in a vertical direction. The isocyanate index was 110. The materials were conditioned for 24 h at room temperature before being cut and tested. The formulations of the polyurethane systems are shown in Table 1.

### 2.3. Methods

Rheological properties of polyol/BioC premixes were investigated. For this purpose, an Anton Paar MCR 301 rotational rheometer equipped with a 25 mm diameter parallel plates system was used. For all measurements, the gap distance of 0.2 mm was constant. In order to evaluate the viscosity of the mixtures, tests were conducted using the constant shear mode, where shear rate was set to 10 s^−1^. In industrial production, the substrates necessary to produce a foam are heated to facilitate the process. Therefore, in our experiment the viscosity measurements were carried out at three temperatures: 30, 50 and 80 °C. 

The foaming process was analyzed using a FOAMAT device (Format-Messtechnik GmbH). The temperature, pressure and dielectric polarization were measured. The changes of temperature during the foaming process were observed using a thermocouple. The dielectric polarization was measured using a Curing Monitor Device (CMD), which gives an insight into electrochemical processes occurring during foam formation. Pressure changes during the foaming process were measured by load of the rising foam on the table (CMD sensor). 

The apparent density was measured as the ratio of the mass and volume of the samples according to ISO 845. 

The thermal conductivity of the polyurethane foams was determined using a Laser Comp heat flow meter constructed according to ISO 8301. An average temperature between two plates was 10 °C. The dimensions of the foams were 200 × 200 × 50 mm^3^. The closed cell content in the foams was measured by the pycnometer method according to the ISO 4590 standard. 

The morphology of the foams was analyzed using an optical microscope (PZO Warszawa), and a scanning electron microscope (Hitachi S-4700). The anisotropy index was calculated as the ratio of the cell heights and widths. 

The compressive strength at 10% deformation was analyzed in accordance with ISO 826. The compressive strength of the foams was measured using a Zwick Z005 TH Allround-Line instrument in two directions, parallel and perpendicular to the rise direction of the foams. 

Dimensional stability was determined according to ISO 2796-1986. The measurement consisted in measuring the changes in the linear dimensions of the samples exposed to a temperature of 70 °C and humidity of 90% for 24 h in an air atmosphere. For testing, 100 × 100 × 25 mm^3^ rigid polyurethane foam samples were measured before and after storage at the specified temperature. 

A thermogravimetric test was conducted in order to determine the decomposition area of the foams. The apparatus used was a Netzsch TG F1 209 Libra. For this purpose, the 10 µm samples were cut out of a foam block and placed inside open ceramic crucibles. Measurements were conducted at temperatures from 30 to 800 °C under a nitrogen purge flow. The heating rate was set to 10 °C/min. The same parameters were used for the analysis of pure BioC filler.

The viscoelastic properties of the foams were determined using the dynamic mechanical thermal analysis technique (DMTA). We used an Anton Paar MCR301 apparatus equipped with torsion mode clamps. Specimens were cut out of foam blocks. The dimensions of a single sample were 10 × 10 × 40 mm. The measurements were conducted in an air atmosphere in the temperature range from 25 to 250 °C, the heating rate was set to 2 °C/min, the deformation amplitude and frequency were 0.1% and 1 Hz, respectively. The DMTA results include the storage modulus, loss modulus and tan δ plots. 

## 3. Results

### 3.1. Rheological Properties

**Figure 3:**
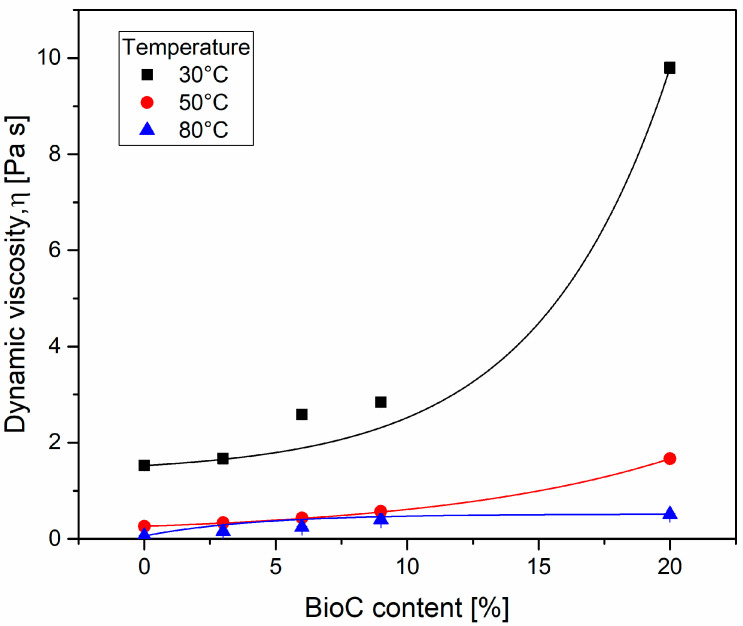
Viscosity of polyol and dispersion of BioC in polyol, measurements at constant shear rate 10 s^−1^.

The results of the constant shear measurement are presented in Figure 3. The dynamic viscosity values were plotted in the graph. The results for individual measurement temperatures were highlighted with separate colors. The horizontal axis corresponds to the BioC content in the final product, while the actual content in the premix is higher, as shown in Table 1. 

As expected, the increasing content of BioC particles visibly increased the viscosity of the polyol premix. The results for the measurement temperature of 30 °C indicated a systematic increase in viscosity, from 1.5 Pa∙s for the polyol sample to 2.8 Pa∙s for the polyol/BioC-9 dispersion. For the highest filler content (polyol/BioC-20), the viscosity reached 9.8 Pa∙s. Such an increase in viscosity can cause potential problems when using static mixers during the industrial polyurethane processing. However, taking into account the results obtained at elevated temperatures (50 and 80 °C), the reduction of viscosity was significant. For example, the viscosity of the polyol/BioC-20 sample at 50 °C was almost equal to that of the pure polyol sample at room temperature. Meanwhile, for the samples tested at 80 °C, the viscosity did not exceed 0.5 Pa·s. Therefore, it can be assumed that technological problems related to the increase in viscosity should not be an obstacle.

### 3.2. Foaming Process and Cell Structure of Rigid Polyurethane Foams with BioC

**Figure 4:**
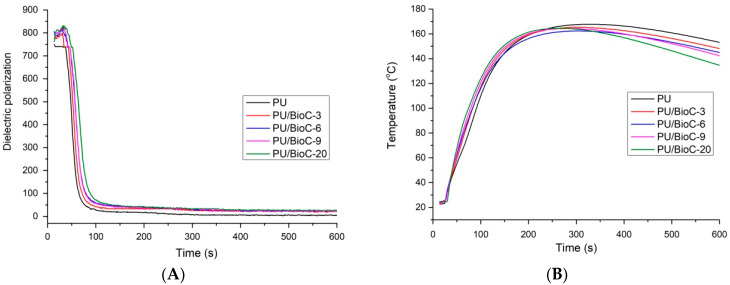

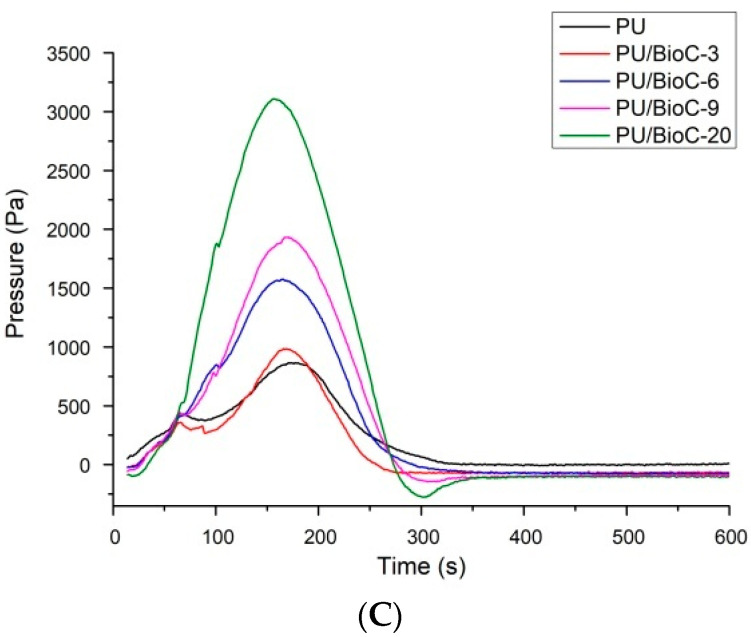
Influence of BioC on dielectric polarization (**A**), temperature (**B**), and pressure (**C**) changes of reaction mixture during foaming of polyurethane systems.

The foaming process is an important step that has an influence on the performance properties of rigid polyurethane foams. The changes of temperature, dielectric polarization and pressure of the polyurethane reaction mixtures used in our experiment during the foaming process are shown in Figure 4. 

The reactivity of the polyurethane system is illustrated by the changes of the dielectric polarization curve. The systems with higher reactivity are characterized by a faster dielectric polarization reduction. Figure 4a shows the dielectric polarization changes during the foaming process for the polyurethane systems modified by BioC. The introduction of BioC as a filler reduced the reactivity of the systems. Figure 4b shows the temperature changes in the core of the foams during the foaming process. Despite a slight reduction in the reactivity of the system, the maximum temperature during the foaming process did not change significantly. The temperature in the foam core was about 160 °C. Additionally, the influence of the filler on the pressure during the foaming process was investigated (Figure 4c). It was observed that with an increasing amount of the filler in the polyurethane system, the pressure inside the foam cells increased. The introduction of the filler into the polyurethane system resulted in an extension of the characteristic foaming times and a slight reduction of maximum temperatures during foaming. A similar effect has been observed in the case of rigid polyurethane foams modified with walnut shells and silanized walnut shells [37]. In our tests, the longer gelation and growth times may have been due to the higher viscosity of the reaction mixture with a higher BioC content. The introduction of the filler into the polyurethane system increased the value of this parameter, further hindering the mobility of molecules and reactions between hydroxyl and isocyanate groups. Increased viscosity of the polyol premix increases the gel time and thus slows down the foaming process [34]. This effect is visible for the system containing 20 wt.% of biochar. Furthermore, lower reactivity of polyurethane systems and the foaming reaction that is faster than the gelling reaction may cause opening of cells in rigid polyurethane foams, as observed by Barczewski et al. [35].

The course of the foaming process influences the formation of the foam cell structure. Table 2 shows the cell characteristics of the resultant foam materials. The addition of BioC to the polyurethane systems reduced the apparent densities of the composites. This is due to the moisture content of the filler used, which was 4.05 wt.%. Moisture in the filler reacted with isocyanate to generate carbon dioxide. A greater amount of a blowing agent reduces the apparent densities of materials [38]. In the case of small additions of fillers, such as carbon fibers and expanded graphite, a decrease in apparent density was also observed [39,40].

It is worth noting that for different types of biochar materials the moisture content can reach even 10%. Hygroscopic nature of BioC is mainly determined by its microporous structure, which, in turn, depends on the type of biomass and the method of pyrolysis. Unfortunately, for most thermoplastics, excessive moisture content leads to the initiation of the phenomenon of hydrolytic degradation, which translates into deterioration of the properties of composites. In the polyurethane production process, the presence of water does not have such a destructive effect, therefore, it is not necessary to eliminate water from the filler. However, the water content should be evaluated and included in the total water content in a polyurethane formulation.

The property that determines the use of a rigid polyurethane foam as an insulation material is its thermal conductivity. The value of thermal conductivity of polyurethane foams depends on many factors including their apparent density, the shape of cells or the type of cells (closed and open) [41]. In our experiment, the introduction of BioC up to 9 wt.% to the rigid polyurethane foams did not change significantly the thermal conductivity coefficient, the value of which was about 25 mW/m·K. However, the addition of BioC in the amount of 20 wt.% increased the value of this parameter. This is due to the increased viscosity of the polyol premix and its inaccurate mixing with the isocyanate. A similar effect was observed for rigid polyurethane foams modified with basalt powder [42]. The introduction of the BioC reduced the apparent density of the foam materials and slightly increased the thermal conductivity. Increasing the thermal conductivity coefficient is a consequence of reducing the content of closed cells in rigid polyurethane foams (Table 2). The results show that the obtained foam materials had closed cell contents in the range of 85–89%.

**Figure 5:**
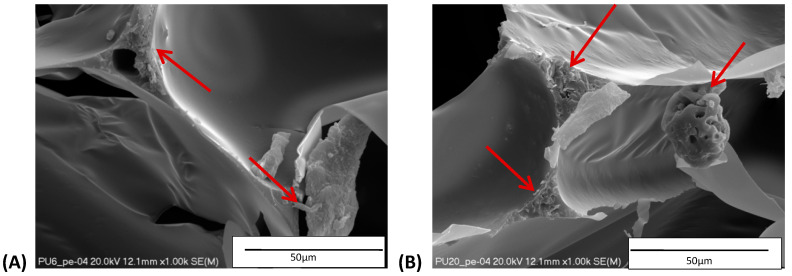
Embedding filler in struts (**A**) (PU/BioC-6), (**B**) (PU/BioC-20). Red arrow shows where the filler is embedded.

Table 2 and Table 3 show parameters of cellular structure and SEM photographs of the foams cross-sections (parallel and perpendicular to the foam growth direction). The anisotropy index is defined as the ratio of cell length to cell width. Cells are assumed to be spherical, if the value of this parameter is 1. The introduction of the filler led to elongation of the cells in a parallel direction, as confirmed by values of the anisotropy coefficient above 1. Cell elongation in the direction of foam growth is related to the initial viscosity of polyurethane systems. Moreover, the application of fillers to a polyurethane system may lead to the nucleation process of the cells of a rigid polyurethane foam [43,44]. This creates more cells while reducing their sizes. In the case of our studies, this effect was also observed—the introduction of BioC resulted in a decrease in the cross-sectional area of the foam cells. The reduction took place in directions both perpendicular and parallel to the foam growth direction. A similar effect has been observed for rigid polyurethane foams modified with expanded graphite [40]. Rigid polyurethane foams consist of 12- and 14-sided structures in the shape of pentagonal dodecahedrons [45]. Between three and more cells there are parts called struts. The filler particles used in our experiment are mostly located in these spaces, which is confirmed by SEM micrographs shown in Figure 5.

### 3.3. Compressive Strength and Dimensional Stability of Rigid Polyurethane Foams with Biochar

**Figure 6:**
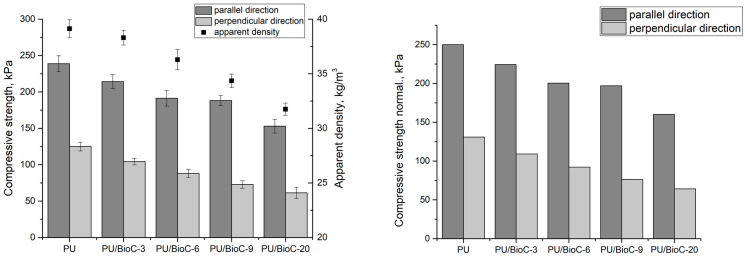
Compressive strength and normalized compressive strength of polyurethane foams.

The mechanical strength depends on the apparent density and cell structure of the foams, i.e., the size and uniformity of the cells [46]. A higher density is associated with more compact cellular structures, hence more material per unit area and a higher strength of the foam material. Figure 6 shows the mechanical strength (σ) and normalized compressive strength (*σ_norm_*) of the BioC-modified foams. Compression experiments were calculated with respect to a density of 40 kg/m^3^. The equation used for the normalization of strength is as follows [47]:

(1) $$\sigma_{norm}=\sigma {\left(\frac{40}{\rho }\right)}^{2.1}$$

Increasing the amount of the filler in the polyurethane systems reduced the compressive strength at 10% deformation. A higher strength was obtained for the foams when measured in a parallel direction to the foam growth direction in contrast to a perpendicular direction. The differences are a result of the anisotropic cell structure of the foams, as confirmed by the data in Table 2 and Table 3. The trend in the increase of the cells anisotropy factor was also reflected in the ratio of the compressive strength at 10% deformation measured in the parallel and perpendicular directions. With a higher amount of the filler in the foams, the apparent density decreased. The lower apparent density resulted in a lower mechanical strength of the foams. This is also due to the lower cross-linking density of these materials. In the literature, it is reported that materials with a higher apparent density are characterized by thicker cell walls and spaces between the foam cells. On the other hand, an introduction of a filler containing water into a polyurethane system resulted in thinner cell walls due to a lower apparent density. Thinner walls and smaller struts lead to a reduction in compressive strength [22]. The filler can also produce more cells that are smaller and irregularly shaped. A disturbance of the cellular structure of a rigid polyurethane foam can also be a cause of a compressive strength reduction.

The changes in the linear dimensions of the reference and modified foams are presented in Table 4.

The introduction of the BioC filler to the polyurethane systems did not cause significant changes in the linear dimensional stability of the materials. The changes in the linear dimensions of the rigid polyurethane foams were less than 1%. Nevertheless, introducing the filler into the systems resulted in changes in the linear dimensions that are smaller than those in the PU reference system. The application of BioC resulted in linearly more stable materials.

**Figure 7:**
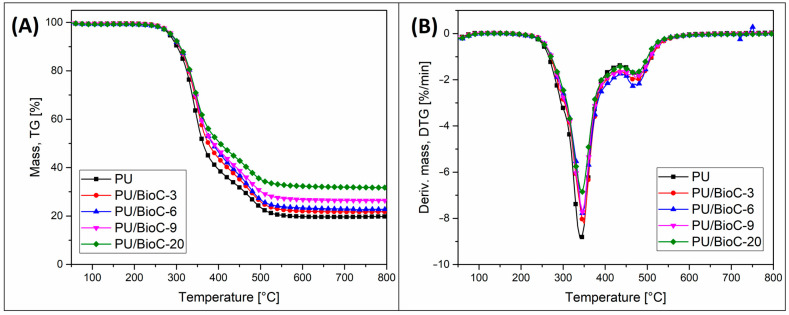
Results of thermogravimetric measurements performed under nitrogen atmosphere. (**A**) TG mass loss plots and (**B**) DTG derivative mass loss plots.

The results of the TGA analysis are presented in the form of TG and DTG plots (see Figure 7). Some of the results, such as DTG peak temperatures, degradation parameters and residual mass, are also collected in Table 5. The addition of BioC particles did not influence significantly the polyurethane decomposition process. The onset of the degradation, usually marked as a 5% weight loss (W 5%), was almost the same for most of the samples. The difference between the pure PU sample (283.4 °C) and PU/BioC-20 (287.6 °C) was only slight. It is worth noting that the thermal stability of the BioC filler is very high, previous research revealed that for similar types of carbonized biomass the weight loss at 800 °C reaches around 15% [9]. Therefore, such a low loss of the filler mass in our tests cannot affect the kinetics of the TGA curves for the composites containing a maximum of 20% of the BioC filler. For the rest of the degradation factors (W 25% and W 50%) the addition of the BioC filler increased the differences for individual samples. For example, the temperature of 50% weight loss for PU/BioC-20 was 40 °C higher than in the case of pure PU, 363.2 and 404.1, respectively. This kind of behavior might suggest an improvement in thermal stability after the addition of BioC, however, a decrease in the kinetics of weight loss is more likely to be associated with the increasing char formation in the PU/BioC compositions. The residual mass for the unmodified PU foam reached around 20%, while for the BioC-modified compositions the weight of residual char was increased, for the PU/BioC-20 sample it reached around 32%. The trend of changes is therefore dependent on the BioC content, however, the increase in the final weight is not closely related to the weight content of the filler.

When considering the overall dynamics of the decomposition process, it is clear that the degradation takes place in two steps, which is more visible in the DTG chart comparison. The first stage related to the degradation of the rigid segments of the polyurethane structure reached the highest intensity at around 340 °C, which is confirmed by the presence of the DTG peak maximum. For the rest of the samples, the DTG peak is only slightly shifted towards higher temperatures. Interestingly, for all of the materials, the second stage of the degradation appears above 400 °C. The DTG peak maximum for this process was detected at around 470 °C for all of the tested samples. This second stage of degradation is associated with the decomposition of the organic residues.

**Figure 8:**
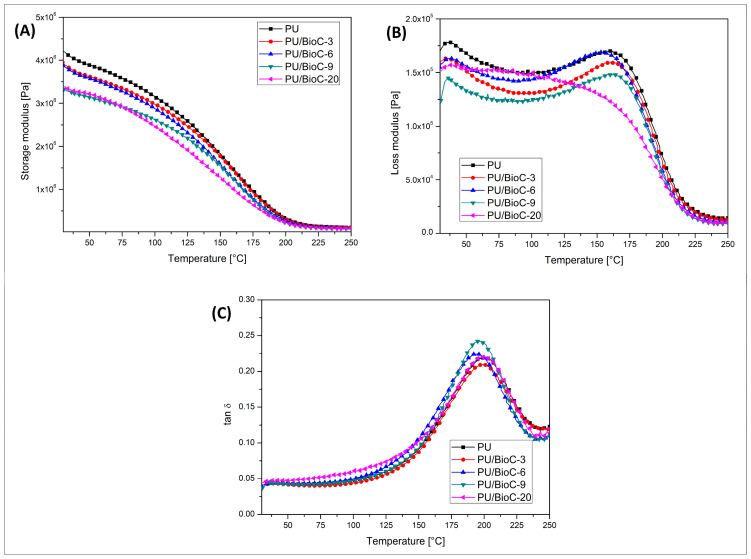
Results of thermomechanical DMTA analysis. (**A**) Storage modulus, (**B**) loss modulus and (**C**) tan δ lots.

The results of the viscoelastic properties evaluation are presented in the form of DMTA plots. The thermograms presented in Figure 8 present the storage modulus, loss modulus and tan δ values. When looking at the storage modulus plots, it is clear that the addition of the BioC particles led to a visible decrease of the polyurethane foam stiffness. The decrease in storage modulus values was visible in the whole range of the DMTA measurement, while it is also clear that the increasing content of the BioC particles led to lower stiffness. The results of the storage modulus evaluation strongly correspond with the results of the compression tests. The use of stiff filler particles did not have any strong effect on the potential strengthening of the material structures. In the case of the foams, the pore formation process and the resulting lower apparent density had a decisive influence on the mechanical properties.

Beside the general differences in the materials stiffness revealed by the storage modulus analysis, it is also possible to compare the damping factor of the materials. Usually, for materials with an addition of spherical particles the damping properties are worse, which is related to the decrease in the value of the tan δ peak. This relationship is also expected in the case of polyurethane foams, as has been confirmed for different types of fillers [43,48]. Interestingly, for the PU/BioC systems prepared in our work, the changes in the damping properties are not related to the filler content. The observed changes in the position of the tan δ peak are very small, even for a sample containing 20 wt.% of the filler. Moreover, these slight changes do not show any visible trend. Summarizing the results of the DMTA analysis, it must be concluded that for the PU/BioC foam systems the increasing content of the filler particles led to a stiffness reduction. Unlike other types of microsized fillers, the reinforcing efficiency of BioC is low. In fact, it is difficult to assess the influence of the filler type, since most of the mechanical properties are strongly related to the foaming process and the apparent density correlation.

## 4. Conclusions

We demonstrated that it is possible to obtain highly effective rigid polyurethane foams with biochar. The introduction of BioC into a polyurethane system affected its initial viscosity and as a consequence the foam formation process. Moreover, the presence of this additional component in the polyurethane system caused a reduction of its reactivity. The changes in the foaming process had an influence on the foam cell structure resulting in cells with smaller cross-sectional areas. Despite lower apparent densities of the foam materials modified with the biochar in an amount of 20 wt.%, their thermal conductivity coefficients did not worsen. Increasing the proportion of the biochar in the polyurethane systems reduced their compressive strengths at 10% strain and the stiffness of the materials, as confirmed by DMTA testing. Biochar in the rigid polyurethane foams improved their dimensional and thermal stability and can be successfully used as filler for polyurethane porous composites. Such materials can be used as thermal insulation boards as well as filling the space between different layers where heat transfer limitation is required.

## Figures and Tables

**Table 1:** Formulations of polyurethane systems.

Component, g	PU	PU/BioC-3	PU/BioC-6	PU/BioC-9	PU/BioC-20
Rokopol RF-551	100.00				
Catalyst	1.50				
Surfactant	1.50				
Water	3.40				
PMDI	169.60				
Biochar (BioC)	0	8.09	16.18	24.26	53.92

**Table 2:** Parameters of cellular structure and physical–mechanical properties of foams.

Name of Sample	Apparent Density, kg/m^3^	Thermal Conductivity, mW/m·K	Percentage of Closed Cells, %	Anisotropy Index		Cross Section Area·10^−2^ mm^2^	
				Parallel	Perpendicular	Parallel	Perpendicular
**PU**	39.1 ± 0.83	25.6 ± 0.26	89.1 ± 1.4	1.26 ± 0.07	0.92 ± 0.02	1.05 ± 0.13	0.74 ± 0.09
PU/BioC-3	38.3 ± 0.66	25. 7 ± 0.11	86.3 ± 1.1	1.30 ± 0.07	0.91 ± 0.02	0.65 ± 0.02	0.42 ± 0.02
PU/BioC-6	36.3 ± 0.93	25.6 ± 0.10	85.4 ± 1.6	1.37 ± 0.03	0.89 ± 0.04	0.71 ± 0.01	0.40 ± 0.04
PU/BioC-9	34.4 ± 0.62	25.7 ± 0.16	87.6 ± 1.1	1.35 ± 0.02	0.89 ± 0.01	0.73 ± 0.01	0.41 ± 0.07
PU/BioC-20	31.8 ± 0.53	27.0 ± 1.21	87.1 ± 2.0	1.50 ± 0.06	0.92 ± 0.02	0.79 ± 0.24	0.46 ± 0.03

**Table 3:** SEM microphotographs of foams.

	PU	PU/BioC-3	PU/BioC-6	PU/BioC-9	PU/BioC-20
**Parallel**	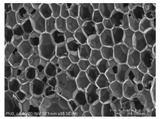	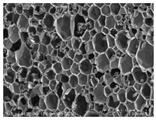	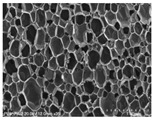	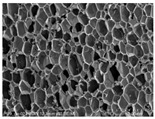	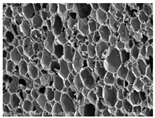
**Perpendicular**	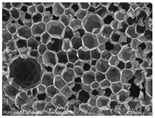	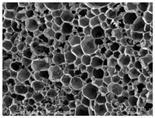	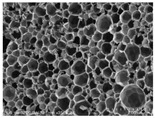	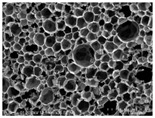	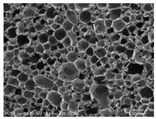

**Table 4:** Dimensional stability of polyurethane foams.

Name of Sample	Dimensional Stability, %		
	Length	Width	Thickness
PU	0.88 ± 0.25	0.59 ± 0.17	−0.16 ± 0.51
PU/BioC-3	0.69 ± 0.58	0.42 ± 0.20	0.12 ± 0.38
PU/BioC-6	0.59 ± 0.08	0.46 ± 0.11	0.05 ± 0.30
PU/BioC-9	0.61 ± 0.11	0.68 ± 0.08	−0.04 ± 0.31
PU/BioC-20	0.57 ± 0.14	0.51 ± 0.12	−0.26 ± 0.31

**Table 5:** Results of TG and DTG plots analysis.

Name of Sample	W 5% (°C)	W 25% (°C)	W 50% (°C)	Residual Mass at 800 °C (%)	DTG Peak Temp. (°C)	DTG Max. Rate (%/min)
PU	283.4	331.6	363.2	20.3	342.3	8.9
PU/BioC-3	287.7	337.0	375.8	22.0	346.9	7.8
PU/BioC-6	287.0	339.5	385.6	22.8	346.7	7.8
PU/BioC-9	288.9	338.6	388.2	26.6	346.4	7.7
PU/BioC-20	287.6	339.8	404.1	31.8	344.1	6.9

## Data Availability

Not applicable.
